# Differential Maize Yield Hybrid Responses to Stand Density Are Correlated to Their Response to Radiation Reductions Around Flowering

**DOI:** 10.3389/fpls.2021.771739

**Published:** 2022-01-17

**Authors:** Federico H. Larrosa, Lucas Borrás

**Affiliations:** ^1^KWS Group, Balcarce, Argentina; ^2^Facultad de Ciencias Agrarias, Universidad Nacional de Rosario, Rosario, Argentina

**Keywords:** corn, stand density, shading treatments, plant population (densities), shading stress

## Abstract

Altered stand density affects maize yields by producing changes in both numerical yield components, kernel number per plant (KNP), and kernel weight (KW). Kernel number is determined by the accumulation of ear biomass during the flowering period, whereas KW is determined by the sink potential established during flowering and the capacity of the plant to fulfill this potential during effective grain filling. Here, we tested if different short shading treatments during different stages around flowering can help discriminate genotypic differences in eco-physiological parameters relevant for maize stand density yield response and associated yield components. Our specific objectives were to: (i) identify hybrids with differential shading stress response, (ii) explore shading effects over eco-physiological parameters mechanistically related to KNP and KW, and (iii) test if shading stress can be used for detecting differential genotypic yield responses to stand density. The objectives were tested using four commercial maize hybrids. Results indicated that KNP was the yield component most related to yield changes across the different shading treatments, and that the specific shading imposed soon after anthesis generated the highest yield reductions. Hybrids less sensitive to shading stress were those that reduced their plant growth rate the least and the ones that accumulated more ear biomass during flowering. Genotype susceptibility to shading stress around flowering was correlated to stand density responses. This indicated that specific shading stress treatments are a useful tool to phenotype for differential stand density responses of commercial hybrids.

## Introduction

Commercial maize breeding programs have been successful in making continuous genetic improvements in maize grain yields (Duvick et al., 2004; Echarte et al., 2004; Luque et al., 2006; Di Matteo et al., 2016; Borrás and Vitantonio-Mazzini, 2018). Hybrid selections are done using multienvironmental trials, where a group of hybrids are grown across several experiments during the years to provide information covering the performance of genotypes in a target population of environments (Delacy et al., 1996). This methodology responds to the requirement of exploring a range of possible environments, with contrasting yield potential and stress conditions. This testing represents a large cost for the production of commercial hybrids. Public and private breeding programs are commonly interested in finding alternative selection methods that allow reducing the number of trials/years during the selection process. Manipulating environmental factors within field experiments can help breeding programs improve genotype selection and agronomic management recommendations, reducing testing costs (Blum and Pnuel, 1990) or increasing their efficiency (Campos et al., 2004).

Environmental conditions, like water (Andrade et al., 2002), nutrient (Caviglia et al., 2014), and radiation levels (Andrade et al., 1999, 2002; Cerrudo et al., 2013) affect maize crop growth and grain yield. Physiological and numeric yield components can help predict crop yield variability associated with different environmental conditions (Rotundo et al., 2012; Di Mauro et al., 2019). Environmental conditions affect maize yields due to changes on kernel number per plant (KNP) or individual kernel weight (KW; Claassen and Shaw, 1970b; Hall et al., 1981). KNP is commonly associated with ear biomass (EB) accumulation around flowering (Echarte et al., 2004; Severini et al., 2011; Borrás and Vitantonio-Mazzini, 2018), and is also associated with plant growth rate (PGR) during this period (Andrade et al., 1999).

Changes in KNP are associated with maize yield variability, and this variability is closely related to changes in PGR around flowering (Fischer and Palmer, 1984; Andrade et al., 1999). Previous studies to determine maize yield susceptibility across the flowering period showed mixed results. Some studies indicated that the close postanthesis period is the most sensitive period for kernel setting and yield (Kiniry and Ritchie, 1985). Other authors, however, indicated that maize yield is most sensitive to changes in canopy growth during 2 weeks bracketing flowering (Hawkins and Cooper, 1981; Cirilo and Andrade, 1994). Otegui and Bonhomme (1998) described the most sensitive period starts −227 Cd (growing degree days) before flowering and ends 100 Cd after flowering, and a recent study found that this period is from −300 to 780 Cd around flowering (Cerrudo et al., 2013).

Several treatments have been used to generate crop stress and reduce canopy growth to test for the crop responses. Yield reductions can be managed using water deficit conditions (Claassen and Shaw, 1970a,b; Hall et al., 1981; Kiniry and Ritchie, 1985; Andrade et al., 2002; Campos et al., 2004), inadequate nutrition (Uhart and Andrade, 1995; Andrade et al., 2002; D’Andrea et al., 2008; Caviglia et al., 2014), increased plant density (Otegui, 1997; Andrade et al., 1999, 2002; Echarte et al., 2000; Sarlangue et al., 2007; Tokatlidis et al., 2011; Hernández et al., 2014), or reductions in radiation levels (Fischer and Palmer, 1984; Kiniry and Ritchie, 1985; Reed et al., 1988; Andrade et al., 1999, 2002). Among all these manipulative stress treatments, artificial shading has practical advantages. The most important is associated with its flexibility for regulating stress timing, intensity, and duration, as shown in the recent study by Cerrudo et al. (2013). Additionally, because canopy growth reductions can be achieved through a number of these treatments, the responses are not specific to the type of stress (Knight and Knight, 2001). In the present manuscript, we tested if genotype differential responses to manipulative shading treatments can be extrapolated to other conditions that reduce canopy growth, like stand density.

Our specific objectives were to: (i) identify tolerant hybrids to shading stress, (ii) explore shading responses using yield numerical and physiological components approaches, and (iii) test if shading stress can be used for detecting differential genotypic yield responses to stand density. We hypothesized that the genotypes with more shading tolerance are the ones with higher optimum stand densities (they tolerate higher stand densities). To test this hypothesis, four maize hybrids were evaluated across different shading and stand density treatments.

## Materials and Methods

### Genotypes

We tested four maize hybrids (H1–H4) with relative maturity between 118 and 125. Genotypes are the result of a single cross between a common female of one heterotic pool and four males with different backgrounds of a second heterotic pool of KWS Group breeding program. Genotype H1 is commercially known as KM3800 (relative maturity 118), H2 is KM4200 (relative maturity 122), H3 is KM4321 (relative maturity 123), and H4 is KM4500 (relative maturity 125).

### Field Experiments With Shading Treatments

Field experiments were conducted in the year 2014 in Zavalla, Santa Fe, Argentina (33° 2′ 24.75″ S, 60° 53′ 11.76″ W). Plots were eight rows with 6 m long and 0.52 m row spacing. Plots were kept free of weeds, insects, and diseases. Weeds were controlled using standard agronomic practices and manually removed whenever necessary. Soil was Vertic Argiudoll, Roldan series. One shading experiment was conducted at Campo Experimental Villarino, Facultad de Ciencias Agrarias, Universidad Nacional de Rosario (named Env 1). Sowing date was September 27 and was conducted under no till and rainfed conditions. All plots were oversown and hand-thinned at V2 (Ritchie and Hanway, 1982) to 8 plants m^–2^. A second shading experiment was conducted at KWS Experimental Station (named Env 2). Sowing date was December 20 and was managed under tillage and rainfed conditions. Plots were hand-planted at three seeds per hill and hand-thinned to one plant per hill at V2 (Ritchie and Hanway, 1982), resulting in a final stand density of 5.5 plants m^–2^.

In both experiments, all the hybrids were shaded during periods of 7 days with 80% reduction of incident photosynthetic active radiation. Five shading treatments were centered around the flowering period. Shading treatments went from 14 to 7 days previous to anthesis (named S−7), from 7 days preanthesis to anthesis (named S0), from anthesis to 7 days after anthesis (named S + 7), and from 7 days after anthesis to 14 after anthesis (named S + 14). A control treatment without shading (named T0) was also evaluated, and in Env 1 an additional treatment starting 21 days before anthesis and ending 14 days before anthesis was also tested (named S−14). The experimental design of both shading experiments was a randomized complete block with three replicates. Shading cloth blocked 80% natural light intensity and was suspended above canopy.

### Field Experiments With Stand Density Treatments

Three additional field experiments testing hybrid response to stand density were conducted at KWS Experimental Station. The three experiments were sown on September 23, October 20, and November 20, 2014, and named Env 3, Env 4, and Env 5, respectively. They included the same four hybrids used in Env 1 and Env 2. Experiments were managed under tillage and rainfed conditions. Plots were four rows with 6 m long and 0.52 m row spacing. Hybrids H1–H4 were tested at four stand densities (5, 7, 9, and 11 plants m^–2^). Plots were oversown and hand-thinned to the desired stand density at V2 (Ritchie and Hanway, 1982). Experiments were fertilized with 40 kg N ha^–1^ before sowing plus an additional 120 kg N ha^–1^ at V6. Plots were kept free of weeds, insects, and diseases. Each stand density experiment had a randomized complete block design of hybrids and stand densities with three replicates.

### Phenotypic Measurements in Shading Experiments

In both shading experiments, yield was calculated from harvesting all ears of two central rows per plot at harvest maturity. Individual kernel weight (KW) was determined after weighting 400 kernels per plot, and KNP was calculated using yield and stand density. Yield and individual KW are reported with 145 g kg^–1^ moisture. Relative yield (RY) was calculated as the ratio between the yield of any shade treatment and the control plot from the same genotype and block.

In the shading experiment of Campo Experimental Villarino, 15 consecutive plants per plot were tagged at V8 in center rows. These plants were used for describing plant growth and kernel number differences across treatments.

At the pre- and postflowering stages, non-destructive allometric models were used to estimate shoot biomass and partitioning at the individual plant level (Vega et al., 2000; Echarte et al., 2004; Gambín et al., 2008). The preflowering model was based on the linear regression between shoot biomass and stem volume (Vega et al., 2001; Gambín et al., 2008). Stem volume was calculated from plant height (ground level up to the uppermost leaf collar) and stem diameter at the base of the stalk. The preflowering biomass sample was done 15 days before 50% anthesis, and the postflowering one was done 15 days after anthesis. In each plot, two plants from border rows were used to develop the allometric preflowering and postflowering models (Vega et al., 2001). All plant samples were determined after cutting plants and drying them in a forced-air oven at 65°C for at least 7 days. The *r*^2^ values for the preflowering model ranged from 0.79 to 0.91 (*p* < 0.01; *n* = 27) across hybrids.

The postflowering biomass sample was done using a multiple linear regression model with stem volume and maximum ear diameter from all ears having extruded visible silks (Vega et al., 2001; Gambín et al., 2008). The *r*^2^ values for this model ranged from 0.81 to 0.98 (*p* < 0.01; *n* = 27) across hybrids. In the postflowering biomass sample, we also estimated the ear biomass 15 days after anthesis by fitting a linear regression between ear biomass and the square of ear diameter (similar to Hernández et al., 2014).

Plant growth rate around flowering (mg plant^–1^°C d^–1^) was calculated as the ratio between the plant biomass (mg plant^–1^) difference and the thermal time accumulated from pre- to postflowering samples in each specific plot. Daily thermal time values were calculated using a base temperature of 8°C. PGR was determined for each tagged plant and the values were presented as an average individual PGR, and its plant-to-plant variability was expressed as the coefficient of variation of PGR (CVPGR) for each genotype × treatment combination.

Barrenness was calculated as the percentage of barren plants per plot. Plants with less than 10 kernels at harvest maturity were considered barren (Tollenaar et al., 1992). For each individual plot, we also calculated the partition efficiency (PEF) as the ratio between EB and the total plant biomass 15 days after anthesis, and the seed set efficiency (SSEF) as the ratio between KNP and the accumulated EB.

**FIGURE 1:**
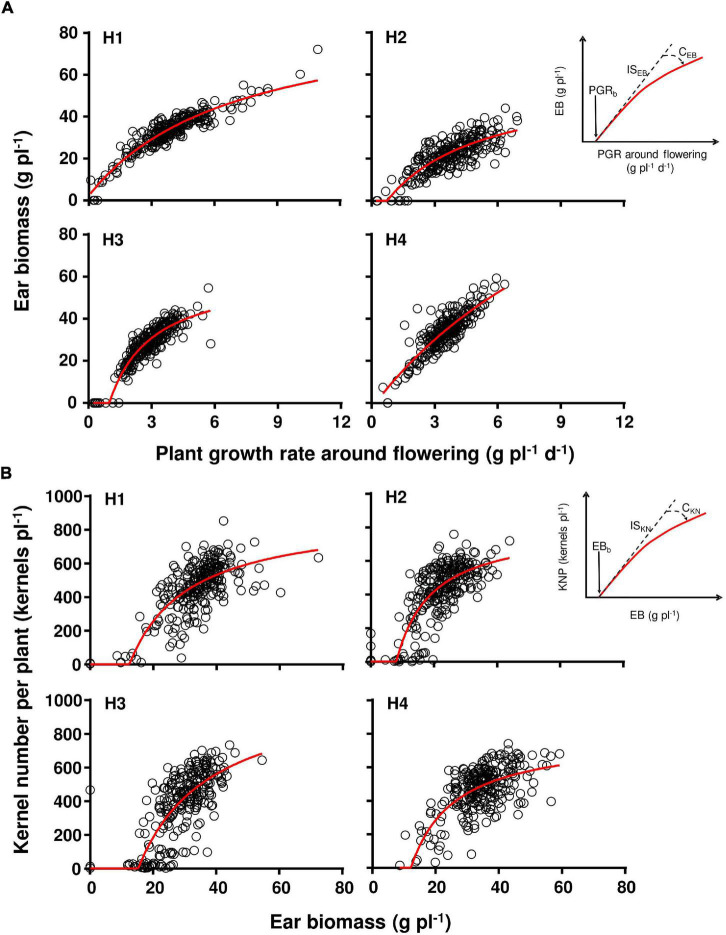
Ear biomass as a function of plant growth rate **(A)**, and kernel number as a function of ear biomass **(B)** for the four evaluated genotypes (H1, H2, H3, and H4). The left inset in **(A,B)** describe the parameters for each relationship, fully described in Table 3. All correlations were significant [**(A)** H1 had *n* = 263, *r*^2^ = 0.54; H2 had *n* = 261, *r*^2^ = 0.59; H3 had *n* = 252, *r*^2^ = 0.55; H4 had *n* = 259, *r*^2^ = 0.45; in **(B)** H1 had *n* = 265, *r*^2^ = 0.88; H2 had *n* = 270, *r*^2^ = 0.65; H3 had *n* = 269, *r*^2^ = 0.84; H4 had *n* = 268, *r*^2^ = 0.75]. Red lines describe the fitted curves.

For comparing hybrids, we fitted the relationship between KNP and EB 15 days after anthesis, and between EB and PGR around flowering, similar to Hernández et al. (2014). Both relationships were described by a hyperbolic function with their specific parameters [Figure 1; Eqs. (1–4)]. Descriptive parameters of the models are PGR_b_, IS_EB_, C_EB_, EB_b_, IS_KN_, and C_KN_ (Figure 1). Models were fit to each genotype × replicate combination and included in the same curve the five shading treatments utilized within each replicate; so the parameters were estimated for genotype × replicate combinations. Replicates were used for an ANOVA test, and *r*^2^ values ranged from 0.41 to 0.92 (*p* < 0.01).


(1) $$\text{EB}=0\;\text{if}\text{PGR}\leq {\text{PGR}}_{\text{b}}$$

(2) $$\text{EB}=\left[{\text{IS}}_{\text{EB}}\times \left(\mathrm{PGR}-{\mathrm{PGR}}_{\text{b}}\right)\right]/[1+{\text{C}}_{\text{EB}}\times \left(\mathrm{PGR}-{\mathrm{PGR}}_{\text{b}}\right)]\;\text{if}\mathrm{PGR}>\mathrm{PGR}{}_{\text{b}}$$

(3) $$\text{KNP}=0\;\text{if}\mathrm{EB}\leq {\mathrm{EB}}_{\text{b}}$$

(4) KNP=[ISKN×(EB-EBb)]/[1+CKN×(EB-EB)b]     ifEB>EBb

where in Eq. (2) IS_EB_ is the initial slope of the relationship between EB and PGR, PGR_b_ is the base PGR for ear growth, and C_EB_ is the curvilinearity of the hyperbolic function (curvature) of the relationship between EB and PGR. In Eq. (4) IS_KN_ is the initial slope of the relationship between KNP and EB, EB_b_ is the base ear biomass for initial kernel set, and C_KN_ is the curvilinearity of the hyperbolic function (curvature) of the relationship between KNP and EB. All these parameters are considered genotypic coefficients. Applying these coefficients uniformly to all the plants is supported by several studies that show a consistent relationship between ear growth and PGR around flowering across environments (Andrade et al., 1999; Vega et al., 2001; Borrás et al., 2007). All curves were fitted using the GraphPad Prism version 5.0 (Raduschev, 2007) iterative optimization technique.

### Phenotypic Measurements in Stand Density Experiments

Yield was calculated after harvesting all ears from 3 m^2^ in central rows at harvest maturity in all genotype × stand density × environment combinations. Yield is reported with 145 g kg^–1^ moisture.

### Statistical Analysis

Data were analyzed separately for each experiment (shading or stand density) in R software (R Core Team, 2020). We used a randomized complete block design with three replications in all the trials. Sources of variation were environment (sowing date), hybrids, treatment (shading or stand density), and blocks. Main or interaction effects were tested with ANOVA. Treatment marginal means were estimated with “emmeans” function from EMMEANS R package (Russell, 2021). Tukey test was done for pairwise comparisons of estimated means.

## Results

### Shading Stress Effects on Maize Yields and Critical Period

Environments, hybrids, and shading treatments all showed significant yield differences (*p* < 0.001; Table 1). A hybrid × shading × environment treatment interaction (*p* < 0.001; Table 1) was also significant, showing that shading stress responses were different among hybrids and environments (*p* < 0.001; Table 1). In both environments, the effects of shading treatments S−14, S + 7, and S + 14 on yield did not differ across hybrids, but large hybrid yield differences were observed in the treatments closer to anthesis (S−7 and S0 treatments; Supplementary Table 1). In Env 1 the S−7 treatment hybrids H1, H2, H3, and H4 yielded 13.0, 11.9, 8.7, and 14.2 Mg ha^–1^, respectively (Supplementary Table 1), and in the S0 treatment genotypes H1, H2, H3, and H4 yielded 11.6, 4.6, 8.2, and 12.7 Mg ha^–1^, respectively (Supplementary Table 1). These genotype differential yield responses were even more evident in Env 2.

**TABLE 1:** Yield, relative yield (yield relative to the T0 control treatment), kernel number per plant (KNP), and individual kernel weight (KW) for four genotypes tested at six reduced radiation treatments (S−14, S−7, S0, S + 7, S + 14, and T0) in two different environments (Env 1 and Env 2).

Environment	Hybrid	Shading	Yield	Relative yield	KNP	Kernel weight
			Mg ha^–1^	%	kernels plant^–1^	mg kernel^–1^
Env 1			12.6	80	433	323
Env 2			8.0	71	401	340
	H1		11.0	80	436	317
	H2		10.0	70	404	329
	H3		10.2	72	386	320
	H4		12.6	86	461	342
		S−14	13.8	91	490	335
		S−7	10.2	77	432	317
		S0	7.1	51	322	343
		S + 7	11.2	85	413	335
		S + 14	11.3	86	401	332
		To	13.2	100	499	316
Env (E)			***(0.5)^¥^	ns	*(23)	***(6)
Hybrid (H)			***(0.9)	***(8)	***(41)	***(12)
Shading (S)			***(1.3)	***(10)	***(57)	***(16)
E × H			ns	*(10)	ns	***(20)
E × S			ns	***(16)	ns	***(24)
H × S			***(3.3)	***(28)	**(150)	*(42)
E × H × S			**(4.8)	*(41)	ns	***(62)

*The S−14 was only tested in Env 1. See section “Materials and Methods” for a description of shading treatments. Treatment mean for interactions are available in Supplementary Table 1.*

^¥^Significance of ***p < 0.001, **p < 0.01, *p < 0.05, and ns is not significant (p > 0.05). Values in parenthesis are Tukey values (p < 0.05).

When analyzed in relative terms (here called relative yield), the interaction environment × hybrid × shading treatment was significant (*p* < 0.05; Table 1), illustrating that yield reductions associated with shading treatments were different depending on the particular hybrid and environment. For example, the relative yield of H1 and H4 in Env 1 did not differ among shading treatments, but the shading treatment finishing at anthesis (S0) applied to H1 had a 26% relative yield, whereas it did not reduce H4 relative yields in Env 2 (Supplementary Table 1, contrast *p* < 0.001). Likewise, hybrids H2 and H3 showed significant relative yield reductions when the shading treatment S0 was applied to both the environments (Supplementary Table 1).

### Shading Effects on Yield Physiological Components

#### Kernel Number per Plant

Kernel number per plant was affected by environment (*p* < 0.05), hybrids (*p* < 0.001), shading (*p* < 0.001), and hybrid × shading treatment interactions (Table 1). This showed that KNP was different according to hybrid and shading treatments (Supplementary Table 1). Hybrid H4 presented the highest KNP values (*p* < 0.001), and when comparing shading treatments S−7, S0, S + 7, and S + 14 significantly lower KNP values were generated in comparison to T0 (*p* < 0.001). Averaged across hybrids, the treatment S0 had the lowest KNP value (322 kernels plant^–1^), in agreement with the described yield response and the known relevance of KNP for yield determination.

Changes in KNP can be described as a function of changes in EB accumulated 15 days after flowering. Significant differences for accumulated EB among hybrids (*p* < 0.001) and shading treatment (*p* < 0.001) were observed. Genotype maximum and minimum EB were 33.7 and 22.1 g ear^–1^, corresponding to H4 and H2, respectively (Table 2). Shading treatments showed maximum values for T0 (32.9 g ear^–1^), and minimum values for S0 (25 g ear^–1^, Table 2).

**TABLE 2:** Kernel number per plant (KNP), accumulated ear biomass 15 days after anthesis (EB), individual plant growth rate around flowering (PGR) and their coefficient of variation (CVPGR), barrenness (BA), partition efficiency (PEF), and seed set efficiency (SSEF) for the four genotypes (H1, H2, H3, and H4) tested at six shading treatments (S−14, S−7, S0, S + 7, S + 14, and T0).

Hybrid	Shading	KNP	EB	PGR	CV_PGR_	BA	PEF	SSEF
		kernels plant^–1^	g ear^–1^	g plant^–1^ d^–1^	%	%	g g^–1^	KNP EB^–1^
H1		456	33.2	3.93	36	3.3	0.21	13.6
H2		425	22.1	3.78	24	7.0	0.13	18.7
H3		383	28.2	2.88	31	8.9	0.20	12.4
H4		468	33.7	3.48	27	3.0	0.21	14.0
	S−14	490	31.2	3.44	25	1.1	0.19	15.7
	S−7	434	26.9	3.31	32	8.3	0.18	15.6
	S0	309	25.0	3.00	30	13.9	0.18	11.6
	S + 7	432	27.9	3.58	32	5.0	0.18	16.4
	S + 14	408	32.0	3.82	27	1.7	0.20	13.0
	T0	525	32.9	3.94	31	3.3	0.19	15.9
Hybrid (H)		^¥^***(50)	***(2.8)	**(0.47)	*(11)	*(5.5)	***(0.01)	***(2.2)
Shading (S)		***(69)	***(3.8)	*(0.64)	ns	***(7.5)	**(0.02)	***(3.0)
H × S		**(178)	ns	Ns	ns	ns	**(0.05)	ns

*See section “Materials and Methods” for a description of the treatments. Treatment mean for interactions are available in Supplementary Table 2.*

^¥^Significance of ***p < 0.001, **p < 0.01, *p < 0.05, and ns is not significant (p > 0.05). Values in parenthesis are Tukey values (p < 0.05).

The proportion of barren plants within the canopy was well correlated to changes in accumulated EB 15 days after anthesis across hybrids and shading treatments, with the S0 treatment being the one with higher barrenness values (Table 2). This was particularly evident in the genotypes H2 and H3 that showed the largest yield and KNP decline in this specific shading treatment, S0 (Supplementary Table 2).

Plant growth rate also showed significant differences for hybrids (*p* < 0.01) and shading treatments (*p* < 0.05; Table 2), and the non-significant hybrid × shading interaction (*p* > 0.05) showed that all the genotypes reduced their growth to a similar extent across shading treatments. Pant growth rate of H1 and H4 was significantly different from H3 (3.93 and 3.48 vs. 2.88 g plant^–1^ day^–1^, respectively; Table 2; contrast *p* < 0.001 and *p* < 0.05, respectively). PGR was significantly reduced for S−14, S−7, and S0 regarding to T0 (Table 2).

Variations in CV_PGR_ only showed significant differences among hybrids (*p* < 0.05, Table 2). H2 was the most uniform genotype, whereas H1 was the most variable one in terms of plant-to-plant growth variability.

Plant biomass partitioning to the ear during the flowering period (called partitioning efficiency, PEF) showed a significant hybrid × shading treatment interaction (*p* < 0.01, Supplementary Table 2). Lowest PEF values were observed for H2, and were especially lower in the shading treatment S0, the one that reduced yield, KNP, and ear biomass accumulation the most.

Also, hybrids (*p* < 0.001) and shading treatments (*p* < 0.01) showed significant differences for their seed set efficiency per unit of accumulated ear biomass (SSEF; Table 2), but no differential hybrid responses were evident for this trait (no significant hybrid × shading interaction, *p* > 0.05; Table 2). The lowest efficiency was observed in the treatment having the highest yield detrimental effect (S0; Table 2).

Figure 1A describes the relationship between ear biomass accumulation and PGR around flowering for each hybrid, and Figure 1B shows the relationship between KNP and ear biomass accumulation for each hybrid. Table 3 describes the parameters of the adjusted models describing the differential response patterns shown by each hybrid. In brief, hybrids showed different response patterns. Parameter PGR_b_ was significantly lower for H1 than H3 (*p* < 0.05, Table 4). When compared with IS_EB_, H3 showed the highest magnitude (31.6 g EB g plant^–1^ d^–1^; *p* < 0.01; Table 4) but was also the genotype with the highest curvature value (C_EB_ for H3 was 0.52 g plant^–1^ d^–1^; Table 3). EB_b_ presented significant differences between hybrids (*p* < 0.05), ranging from 9.2 to 16.5 g (Table 3), and the lowest value was observed in H2. As such, genotypes differed in the parameters that described how much of the total plant biomass is partitioned to the growing ear around flowering, and in how is accumulated ear biomass turned into kernels per plant. The poor plant biomass partitioning described in H2 in Table 2 is also evident in Figure 1 and is coincident with the hybrid susceptibility to shading.

**TABLE 3:** Descriptive parameters of model relating kernel number per plant (KNP) with ear biomass accumulated 15 days after anthesis (EB), and EB as a function of plant growth rate (PGR) around flowering.

Hybrid	PGR_b_	IS_EB_	C_EB_	EB_b_	IS_KN_	C_KN_
	g plant^–1^ d^–1^	g EB g plant^–1^ d^–1^	g plant^–1^ d^–1^	g	KNP g EB^–1^	g EB
H1	0.13	14.5	0.17	11.2	40.8	0.04
H2	0.52	11.9	0.20	9.2	105.0	0.15
H3	1.00	31.6	0.52	16.5	58.0	0.06
H4	0.24	13.3	0.08	12.2	56.1	0.07
Hybrid	*(0.85)	**(14.5)^¥^	*(0.40)	*(6.6)	ns	ns

*PGR_b_ is the minimum base plant growth rate around flowering for ear biomass accumulation, IS_EB_ is the initial slope of the relationship between plant growth rate and ear biomass accumulated at 15 days after anthesis, C_EB_ is the curvature of the relationship between ear biomass and plant growth rate, EB_b_ is the base ear biomass around flowering for kernel number per plant, IS_KN_ is the initial slope of the ear biomass vs. kernel number per plant relationship, and C_KN_ is the curvature of the relationship between ear biomass and kernel number per plant relationship. This is described for four genotypes (H1, H2, H3, and H4). Additional data available in the section “Materials and Methods.”*

^¥^Significance of ***p < 0.001, **p < 0.01, *p < 0.05, and ns is not significant (p > 0.05). Values in parenthesis are Tukey values (p < 0.05).

**TABLE 4:** Yield of four genotypes (H1, H2, H3, and H4) tested at three environments (Env 3, Env 4, and Env 5), and four stands density treatments (D1, D2, D3, and D4 were 5, 7, 9, and 11 plants m^–2^).

Environment	Hybrid	Stand density	Yield
			Mg ha^–1^
Env 3			14.1
Env 4			13.9
Env 5			12.3
	H1	D1	10.9
		D2	13.8
		D3	14.1
		D4	14.0
	H2	D1	11.7
		D2	13.6
		D3	14.0
		D4	12.9
	H3	D1	11.9
		D2	15.0
		D3	13.8
		D4	13.0
	H4	D1	12.4
		D2	15.0
		D3	14.9
		D4	14.2
Environment (E)			***(0.5)^¥^
Hybrid (H)			***(0.6)
Stand density (SD)			***(0.6)
H × SD			*(1.6)
E × H			*(1.3)
E × SD			ns
E × H × SD			ns

*Treatment mean for interactions are available in Supplementary Table 3.*

^¥^Significance of ***p < 0.001, **p < 0.01, *p < 0.05, and ns is not significant (p > 0.05). Values in parenthesis are Tukey values (p < 0.05).

### Hybrid Differential Yield Response to Stand Density

In a second round of experiments, we tested how these same hybrids responded to stand density changes, and a stand density × hybrid experiment was repeated across three environments.

Yield results showed that all three main effects (hybrids, stand densities, and environments) were highly significant (*p* < 0.001), and that the interactions hybrid × stand density and hybrid × environment were also statistically significant for yield (*p* < 0.05; Table 4). The significant interaction hybrid × stand density showed that hybrids responded differently to changes in stand density.

Analyzing hybrids across densities, H4 produced highest yields in all densities, yielding 12.4, 15.0, 14.9, and 14.2 Mg ha^–1^ for stand densities 5, 7, 9, and 11 plants m^–2^, respectively. Hybrid H1 also presented its highest yields in the highest stand densities, with 14.1 and 14.0 Mg ha^–1^, at 9 and 11 plants m^–2^, respectively (Table 4). Contrary to this response, hybrids H2 and H3 did not maximize their yields at the highest densities and showed a significant decline in their yields at the highest stand density of 11 plants m^–2^. Hybrids H2 and H3 showed that the maximum yields were achieved at the lower stand densities of 9 and 5 plants m^–2^, respectively (Table 4). This differential yield response to stand the density of hybrids H2 and H3 compared to H1 and H4 was more evident in the lowest yield environment Env 5 (Supplementary Table 3).

## Discussion

Differences among commercial maize hybrids in their yield response to water availability (Campos et al., 2004; Messina et al., 2019), stand density (Sarlangue et al., 2007; Tokatlidis et al., 2011; Hernández et al., 2014; Mylonas et al., 2020), and *N* availability (Gambin et al., 2016) are known. Crop managers are seeking information about hybrid × stand density interactions, and many seed companies are currently providing hybrid-specific recommendations for stand density management. The generation of this information comes with a large effort, in which commercial and precommercial hybrids are tested at a range of stand densities and environments to provide accurate recommendations (Lacasa et al., 2020).

In the present manuscript, we tested the hypothesis that hybrid response to shading treatments around flowering can help predict hybrid differential responses to stand density. This hypothesis is based on the concept that all these stressful environmental scenarios (lack of water, of radiation, of N) have common responses affecting kernel set though changes in PGR around the flowering period (Andrade et al., 1999). We do realize that our study tested a limited number of hybrids, locations, and stand densities, but results have large implications for phenotyping hybrid responses to management changes. Our results testing a number of commercial genotypes support the use of specific shading treatments to predict hybrid stand density performance.

We tested five different short shading moments to identify if there was any specific timing that helped discriminate hybrids in their response to shading. Results indicated that the shading ending at anthesis (S0) was the most powerful one to discriminate differential hybrid responses to shading stress. The treatments that were more distanced in time from anthesis (starting 14 days before or after anthesis, treatments S−14 and S + 14, respectively) were the ones showing minor yield effects. This is coincident with the early articles about the maize yield critical period around flowering (Fischer and Palmer, 1984; Otegui and Bonhomme, 1998), and contradicts the results from Cerrudo et al. (2013) that predicted a similar effect for a large period around flowering. We hypothesize that differences with this later study might be a consequence of testing a single genotype, our hybrid × shading significant interaction for yield (Table 1) shows that not all genotypes have similar yield responses.

Maize grain yield response to stand density changes is usually dissected into two components, potential yield per plant, and tolerance to crowding stress. Although evidence is available that both components have changed with breeding for yield, the latter component has been more successfully increased by breeding and is responsible for the most yield improvements (Tollenaar and Wu, 1999; Duvick et al., 2004; Tokatlidis and Koutroubas, 2004; Egli, 2015; Assefa et al., 2018). In the present manuscript, we described that a direct specific shading treatment around flowering can help predict hybrid performance to higher stand densities, as shown by the differential response of the commercial evaluated hybrids.

Amelong et al. (2017) reported that hybrid yield response to stand density can be predicted from parental inbred line information. In the present study, we used four genotypes that only differed in one parental line. This will allow us to track the genotypic basis for the differential shading, and stand density described yield responses.

## Conclusion

Evaluated hybrids differ in their yield and relative yield response to changes in shading stress. The treatments that exposed hybrid differences the most were those specifically centered around the flowering period.

Yield responses to shading stress were related to known physiological determinants of kernel set, namely plant growth and biomass partitioning to the ear during flowering. These physiological traits helped understand commercial hybrid differences in their yield sensitivity to shading stress.

Hybrid differences in their yield response to high stand density were correlated to their yield response to shading stress, indicating that shading treatments can be used to effectively test hybrid yield performance to crowing tolerance.

## Data Availability Statement

The raw data supporting the conclusions of this article will be made available by the authors, without undue reservation.

## Author Contributions

Both authors listed have made a substantial, direct and intellectual contribution to the work, and approved it for publication.

## Conflict of Interest

FL was employed by company KWS Group. The remaining authors declare that the research was conducted in the absence of any commercial or financial relationships that could be construed as a potential conflict of interest.

## Publisher’s Note

All claims expressed in this article are solely those of the authors and do not necessarily represent those of their affiliated organizations, or those of the publisher, the editors and the reviewers. Any product that may be evaluated in this article, or claim that may be made by its manufacturer, is not guaranteed or endorsed by the publisher.

## Abbreviations

EB: ear biomass
PGR: plant growth rate
KNP: kernel number per plant
KW: kernel weight
RY: relative yield
CV_PGR_: coefficient of variation of plant growth rate
BA: barrenness
PEF: partition efficiency
SSEF: seed set efficiency
IS_EB_: initial slope for ear biomass accumulation
PGR_b_: base plant growth rate for ear biomass accumulation
C_EB_: curvilinearity of the relationship between ear biomass accumulation and plant growth rate
IS_KN_: initial slope for kernel number per plant
EB_b_: base ear biomass for kernel set
C_KN_: curvilinearity of the relationship between kernel number per plant and accumulated ear biomass
Env: environment
H: hybrids
S: shading
SD: stand density.
