# Supplementary material for: Differential Maize Yield Hybrid Responses to Stand Density Are Correlated to Their Response to Radiation Reductions Around Flowering
Source: Front Plant Sci. 2022 Jan 17;12:771739. doi: 10.3389/fpls.2021.771739 (PMC8801883; doi:10.3389/fpls.2021.771739)
Supplement: Supplementary file 1 [file Data_Sheet_1.docx]

**Supplementary tables**

**Table S1**: Yield, relative yield (treatment shading yield / treatment control yield), kernel number per plant (KNP), kernel weight (KW) for four genotypes (H1, H2, H3, and H4) tested at six shading treatments (S-14, S-7, S0, S+7, S+14, and T0) in two different environments (Env 1 and Env 2).The S-14 was only tested in Env 1.

| Hybrid | | Shading |  |  | Env 1 | |  |  |  | Env 2 |  |
| --- | --- | --- | --- | --- | --- | --- | --- | --- | --- | --- | --- |
|  | |  | Yield | Relative  yield | KNP | | Kernel  weight | Yield | Relative  Yield | KNP | Kernel  weight |
|  | |  | Mg ha^-1^ | % | kernels  plant^-1^ | | mg kernel^-1^ | Mg ha^-1^ | % | kernels  plant^-1^ | mg kernel^-1^ |
|  | |  |  |  |  | |  |  |  |  |  |
| H1 | | Average | 12,5 | 88 | 456 | | 295 | 8,2 | 76 | 398 | 338 |
|  | | S-14 | 13,1 | 92 | 511 | | 285 | - | - | - | - |
|  | | S -7 | 13,0 | 92 | 501 | | 287 | 7,3 | 68 | 401 | 333 |
|  | | S0 | 11,6 | 81 | 363 | | 288 | 2,8 | 26 | 301 | 333 |
|  | | S+7 | 11,2 | 79 | 410 | | 300 | 10,7 | 100 | 400 | 344 |
|  | | S+14 | 11,9 | 84 | 400 | | 309 | 9,5 | 88 | 447 | 344 |
|  | | T0 | 14,3 | - | 554 | | 299 | 10,7 | - | 441 | 336 |
|  | |  |  |  |  | |  |  |  |  |  |
| H2 | | Average | 11,9 | 79 | 425 | | 326 | 6,5 | 69 | 365 | 334 |
|  | | S-14 | 14,1 | 94 | 451 | | 306 | - | - | - | - |
|  | | S-7 | 11,9 | 78 | 433 | | 326 | 7,7 | 81 | 413 | 297 |
|  | | S0 | 4,6 | 31 | 230 | | 302 | 0,8 | 8 | 242 | 425 |
|  | | S+7 | 13,6 | 90 | 490 | | 345 | 6,7 | 71 | 336 | 317 |
|  | | S+14 | 12,4 | 82 | 424 | | 347 | 7,8 | 83 | 386 | 333 |
|  | | T0 | 15,0 | - | 524 | | 323 | 9,7 | - | 445 | 297 |
|  | |  |  |  |  | |  |  |  |  |  |
| H3 | | Average | 11,9 | 80 | 383 | | 324 | 7,2 | 71 | 371 | 321 |
|  | | S-14 | 13,5 | 91 | 447 | | 324 | - | - | - | - |
|  | | S-7 | 8,7 | 58 | 316 | | 314 | 6,2 | 61 | 400 | 322 |
|  | | S0 | 8,2 | 56 | 210 | | 302 | 2,1 | 20 | 248 | 344 |
|  | | S+7 | 12,7 | 86 | 407 | | 352 | 9,1 | 90 | 381 | 319 |
|  | | S+14 | 13,2 | 89 | 405 | | 331 | 8,4 | 83 | 360 | 331 |
|  | | T0 | 14,9 | - | 512 | | 323 | 10,2 | - | 467 | 286 |
|  | |  |  |  |  | |  |  |  |  |  |
| H4 | | Average | 14,1 | 86 | 468 | | 336 | 9,9 | 93 | 450 | 366 |
|  | | S-14 | 14,2 | 88 | 552 | | 335 | - | - | - | - |
|  | | S-7 | 14,2 | 87 | 488 | | 322 | 9,6 | 90 | 500 | 363 |
|  | | S0 | 12,7 | 78 | 434 | | 317 | 9,3 | 86 | 465 | 394 |
|  | | S+7 | 12,5 | 77 | 421 | | 343 | 10,5 | 97 | 421 | 386 |
|  | | S+14 | 14,5 | 89 | 402 | | 367 | 9,3 | 89 | 374 | 350 |
|  | | T0 | 16,2 | - | 508 | | 329 | 10,9 | - | 493 | 339 |
|  |  | |  |  | |  |  |  |  |  |  |

**Table S2**: Day of anthesis, accumulated ears biomass 15 days after anthesis (EB), individual plant growth rate around flowering (PGR) and their coefficient of variation (CVPGR), barrenness, partition efficiency, and seed set efficiency for four genotypes tested withsix shading treatments (six shading treatments (S-14, S-7, S0, S+7, S+14, and T0).

| Hybrid | Shading |  | | EB | PGR | CV PGR | Barrenness | Partition  EF | | SSEF |  |
| --- | --- | --- | --- | --- | --- | --- | --- | --- | --- | --- | --- |
|  |  |  | | g plant^-1^ | g plant^-1^ d^-1^ | % | % |  |  | | |
|  |  |  | |  |  |  |  |  |  | | |
| H1 | Average |  | | **33,2** | **3,93** | **36** | **3,3** | **0,21** | **13,6** | | |
|  | S-14 |  | | 34,5 | 3,92 | 28 | 4,4 | 0,21 | 14,7 | | |
|  | S -7 |  | | 32,9 | 3,83 | 24 | 2,2 | 0,21 | 14,8 | | |
|  | S0 |  | | 29,8 | 3,31 | 40 | 2,2 | 0,22 | 11,6 | | |
|  | S+7 |  | | 30,3 | 3,71 | 49 | 6,7 | 0,21 | 14,5 | | |
|  | S+14 |  | | 35,2 | 4,21 | 30 | 2,2 | 0,21 | 11,4 | | |
|  | T0 |  | | 36,7 | 4,57 | 44 | 2,2 | 0,19 | 14,6 | | |
|  |  |  | |  |  |  |  |  |  | | |
| H2 | Average |  | | **22,1** | **3,78** | **24** | **7,0** | **0,13** | **18,7** | | |
|  | S-14 |  | | 24,6 | 3,51 | 21 | - | 0,16 | 18,3 | | |
|  | S-7 |  | | 19,3 | 3,61 | 25 | 13,3 | 0,12 | 21,1 | | |
|  | S0 |  | | 15,0 | 2,87 | 27 | 22,2 | 0,10 | 13,7 | | |
|  | S+7 |  | | 21,6 | 4,36 | 23 | 4,4 | 0,12 | 22,9 | | |
|  | S+14 |  | | 25,8 | 4,18 | 24 | - | 0,15 | 16,6 | | |
|  | T0 |  | | 26,4 | 4,12 | 26 | 2,2 | 0,15 | 19,7 | | |
|  |  |  | |  |  |  |  |  |  | | |
| H3 | Average |  | | **28,2** | **2,88** | **31** | **8,9** | **0,20** | **12,4** | | |
|  | S-14 |  | | 29,2 | 2,85 | 35 | - | 0,20 | 14,5 | | |
|  | S -7 |  | | 26,3 | 2,65 | 34 | 13,3 | 0,18 | 10,0 | | |
|  | S0 |  | | 23,2 | 2,49 | 35 | 24,4 | 0,17 | 7,4 | | |
|  | S+7 |  | | 26,9 | 2,75 | 33 | 6,7 | 0,20 | 14,9 | | |
|  | S+14 |  | | 30,9 | 3,28 | 24 | 4,4 | 0,20 | 12,7 | | |
|  | T0 |  | | 32,9 | 3,28 | 26 | 4,4 | 0,21 | 15,1 | | |
|  |  |  | |  |  |  |  |  |  | | |
| H4 | Average |  | | **33,7** | **3,48** | **27** | **3,0** | **0,21** | **14,0** | | |
|  | S-14 |  | | 36,4 | 3,48 | 17 | - | 0,21 | 15,4 | | |
|  | S -7 |  | | 29,4 | 3,15 | 44 | 4,4 | 0,19 | 16,4 | | |
|  | S0 |  | | 31,8 | 3,33 | 18 | 6,7 | 0,21 | 13,8 | | |
|  | S+7 |  | | 33,0 | 3,51 | 25 | 2,2 | 0,20 | 13,1 | | |
|  | S+14 |  | | 36,1 | 3,61 | 31 | - | 0,22 | 11,3 | | |
|  | T0 |  | | 35,7 | 3,78 | 28 | 4,4 | 0,22 | 14,1 | | |
|  | | |  |  |  |  |  |  |  | | |

**Table S3**: Differences in yield for four hybrids (H1, H2, H3, and H4) tested at three environments(Env 3, Env 4, and Env 5) and four stands density treatments (D1, D2, D3, and D4 were 5, 7, 9, and 11 plants m^2^, respectively).

| Environment | Hybrid | | Stand density | Yield |
| --- | --- | --- | --- | --- |
|  |  | |  | Mg ha^-1^ |
|  |  | |  |  |
| Env 3 | H1 | | D1 | 11,3 |
|  |  | | D2 | 14,6 |
|  |  | | D3 | 14,4 |
|  |  | | D4 | 14,3 |
|  |  | |  |  |
|  | H2 | | D1 | 12,9 |
|  |  | | D2 | 14,1 |
|  |  | | D3 | 15,5 |
|  |  | | D4 | 13,9 |
|  |  | |  |  |
|  | H3 | | D1 | 12,6 |
|  |  | | D2 | 15,8 |
|  |  | | D3 | 14,9 |
|  |  | | D4 | 13,2 |
|  |  | |  |  |
|  | H4 | | D1 | 12,2 |
|  |  | | D2 | 15,9 |
|  |  | | D3 | 15,4 |
|  |  | | D4 | 14,4 |
|  |  | |  |  |
| Env 4 | H1 | | D1 | 11,2 |
|  |  | | D2 | 14,2 |
|  |  | | D3 | 14,6 |
|  |  | | D4 | 14,3 |
|  |  | |  |  |
|  | H2 | | D1 | 11,9 |
|  |  | | D2 | 13,8 |
|  |  | | D3 | 14,1 |
|  |  | | D4 | 13,6 |
|  |  | |  |  |
|  | H3 | | D1 | 12,6 |
|  |  | | D2 | 16,0 |
|  |  | | D3 | 14,6 |
|  |  | | D4 | 14,2 |
|  |  | |  |  |
|  | H4 | | D1 | 12,7 |
|  |  | | D2 | 15,2 |
|  |  | | D3 | 15,2 |
|  |  | | D4 | 15,2 |
|  |  | |  |  |
| Env 5 | H1 | | D1 | 10,2 |
|  |  | | D2 | 12,7 |
|  |  | | D3 | 13,4 |
|  |  | | D4 | 13,6 |
|  |  | |  |  |
|  | H2 | | D1 | 10,4 |
|  |  | | D2 | 12,8 |
|  |  | | D3 | 12,3 |
|  |  | | D4 | 11,3 |
|  |  | |  |  |
|  | H3 | | D1 | 10,6 |
|  |  | | D2 | 13,2 |
|  |  | | D3 | 11,8 |
|  |  | | D4 | 11,7 |
|  |  | |  |  |
|  | H4 | | D1 | 12,4 |
|  |  | | D2 | 14,0 |
|  |  | | D3 | 14,0 |
|  |  | | D4 | 13,0 |
|  |  |  | |  |
